# Leveraging systems science and design thinking to advance implementation science: moving toward a solution-oriented paradigm

**DOI:** 10.3389/fpubh.2024.1368050

**Published:** 2024-05-15

**Authors:** Terry T.-K. Huang, Emily A. Callahan, Emily R. Haines, Cole Hooley, Dina M. Sorensen, David W. Lounsbury, Nasim S. Sabounchi, Peter S. Hovmand

**Affiliations:** ^1^Center for Systems and Community Design and NYU-CUNY Prevention Research Center, Graduate School of Public Health & Health Policy, City University of New York, New York, NY, United States; ^2^EAC Health and Nutrition, LLC, Leesburg, VA, United States; ^3^School of Medicine, Wake Forest University, Winston-Salem, NC, United States; ^4^School of Social Work, Brigham Young University, Provo, UT, United States; ^5^d.studio, Charlotte, NC, United States; ^6^Department of Epidemiology & Population Health, Albert Einstein College of Medicine, Bronx, NY, United States; ^7^Center for Systems and Community Design, Graduate School of Public Health & Health Policy, City University of New York, New York, NY, United States; ^8^Center for Community Health Integration, School of Medicine, Case Western Reserve University, Cleveland, OH, United States

**Keywords:** systems science, design thinking, implementation science, human-centered design, complex systems, prevention, intervention sustainability, intervention scale

## Abstract

Many public health challenges are characterized by complexity that reflects the dynamic systems in which they occur. Such systems involve multiple interdependent factors, actors, and sectors that influence health, and are a primary driver of challenges of insufficient implementation, sustainment, and scale of evidence-based public health interventions. Implementation science frameworks have been developed to help embed evidence-based interventions in diverse settings and identify key factors that facilitate or hinder implementation. These frameworks are largely static in that they do not explain the nature and dynamics of interrelationships among the identified determinants, nor how those determinants might change over time. Furthermore, most implementation science frameworks are top-down, deterministic, and linear, leaving critical gaps in understanding of both how to intervene on determinants of successful implementation and how to scale evidence-based solutions. Design thinking and systems science offer methods for transforming this problem-oriented paradigm into one that is solution-oriented. This article describes these two approaches and how they can be integrated into implementation science strategies to promote implementation, sustainment, and scaling of public health innovation, ultimately resulting in transformative systems changes that improve population health.

## 1 Introduction

Many persistent public health challenges are marked by dynamic complexity that makes them particularly difficult for practitioners and policymakers to address (1). A key characteristic of such problems—for example, rising prevalence of obesity (2) and widespread opioid addiction and dependence (3)—is involvement of multiple heterogenous factors, actors, and sectors that affect relevant behaviors and health outcomes. Additional key characteristics include interconnectivity and interdependencies with other problems; persistence over time and adaptation to changing circumstances; high economic and/or political stakes; and lack of agreement or clarity regarding solutions (4).

This inherent complexity is a primary driver of the present challenges of insufficient implementation, sustainment, and scale of evidence-based public health interventions. It has been estimated that about half of available public health innovations are used in practice, (5) and that it takes 17 years for just 14% of evidence-based research outcomes to be implemented in real-world settings (6, 7). Moreover, many evidence-based public health interventions are seldom sustained, with limited funding and resources identified as a primary barrier (8).

A goal of implementation science for public health is to identify the factors, processes, and methods that can successfully embed evidence-based interventions in policy and practice, hastening the translation from discovery to application and population health benefits (9). Multiple implementation science frameworks have been developed to offer strategies to generalize findings across diverse settings, identify implementation determinants (e.g., contextual barriers and facilitators), inform data collection, enhance conceptual clarity, and guide implementation planning (10). Identifying key factors that facilitate or hinder implementation is beneficial, but many of the frameworks are static in that they do not explain the nature and dynamics of interrelationships among the identified determinants nor how those determinants might change over time. Many implementation science frameworks are top-down and deterministic (11), although some recent advances have been made to address community engagement in intervention implementation (12) and to guide adaptations during program implementation (13). Most implementation science frameworks, however, do not necessarily pinpoint the most promising focus areas or determinants, let alone *how* to intervene on them (i.e., how to select, design, tailor, and deliver implementation strategies) (14).

Design thinking and systems science offer methods for transforming a traditionally problem-oriented paradigm into one that is solution-oriented. We propose that integrating these two approaches into implementation science strategies can promote implementation, sustainment, and scaling of public health innovation, ultimately resulting in transformative systems changes that improve population health. Herein we describe how design thinking and systems science can be leveraged to advance implementation science, first by describing each approach and its benefits and then discussing how the two can be integrated in implementation science approaches to fill gaps.

## 2 Innovating through design thinking

**Figure 1:**
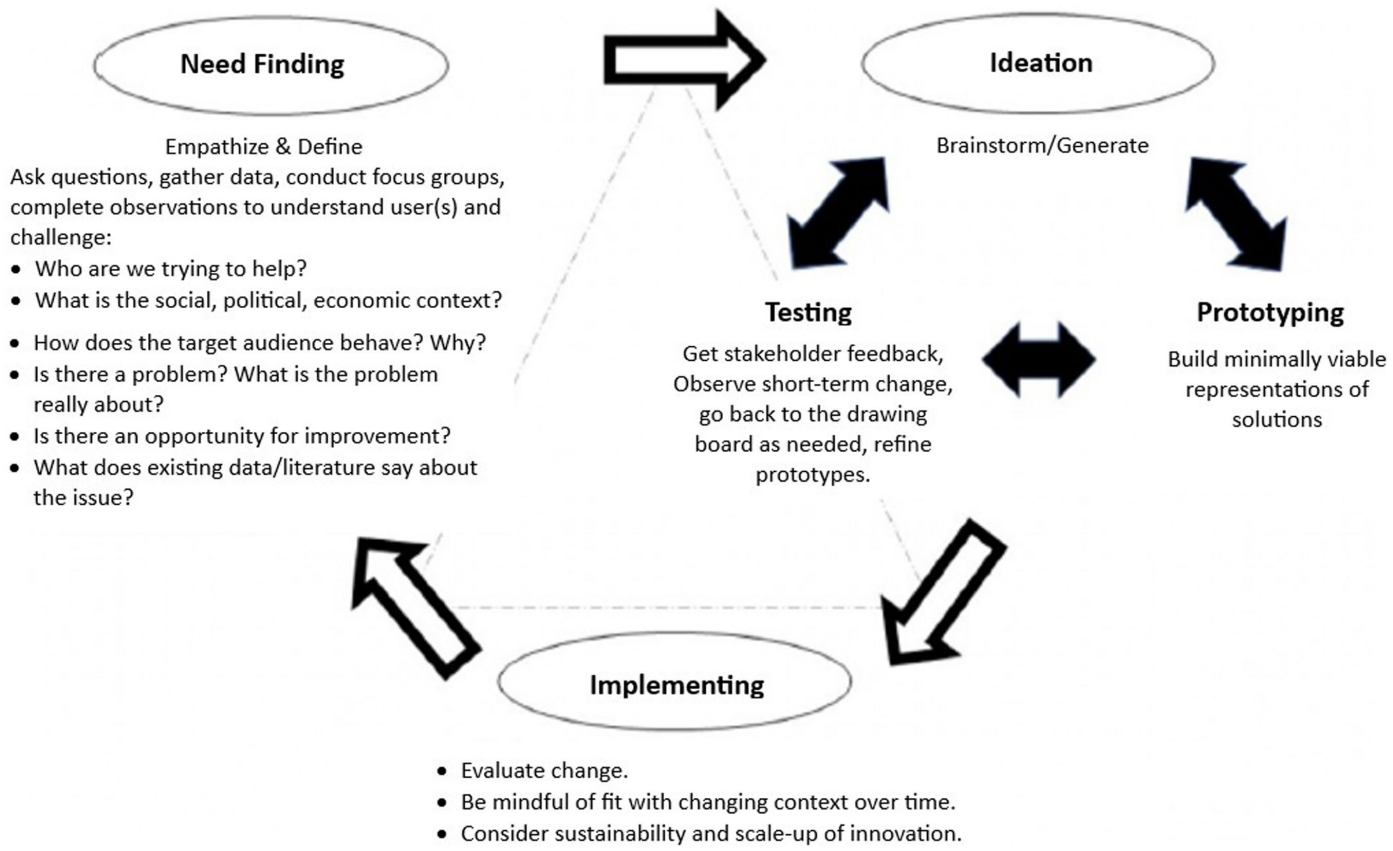
A process model of design thinking. Source: adapted from Altman et al. (16), https://www.cdc.gov/pcd/issues/2018/18_0128a.htm#1.

Design thinking is a human-centered approach to innovation that includes methods from the designer’s toolkit to solve problems through creativity. A design thinking approach embraces complexity and provides a methodologically deliberate, non-linear way of developing an understanding of and structural empathy (15) with the populations affected by an issue. It reframes an issue to generate new ideas or surprising solutions by rapidly prototyping and testing the ideas and learning from them in an iterative manner (16). Key stages in the design thinking process have been summarized as moving from problem identification to design challenge opportunity by empathizing, defining, ideating, prototyping, and testing new solutions for refinement. The process is ultimately action-oriented and focused on *solving* implementation challenges identified by current implementation science frameworks. Figure 1 highlights stages in the design thinking process and lists exercises used or questions asked in each stage.

These dynamic stages can be performed multiple times as the design challenge solution is iterated through experimentation, testing, and refining of ideas toward a feasible solution. Because the process emphasizes rapid and iterative ideation, prototyping, and testing of solution ideas in an environment where it is safe—and even encouraged—to repeatedly fail, lessons learned about what leads to failure can be rapidly parlayed into new and improved solutions ideas (14). For example, a Danish project that investigated how health professionals practice shared decision-making with cancer patients was successful in using design thinking methods to better understand patients’ needs before, during, and after treatment and in prototyping novel potential solutions for supporting and empowering patients during their treatment (17). In another case, a human-centered design approach was used to improve feasibility and acceptability of an HIV response intervention that promoted patient-centered care practices among health care workers in Zambia (18).

The design thinking process can also generate insights about usability and fit of evidence-based community health strategies based on user needs and contexts, as well as other implementation factors such as feasibility and complexity (19). For example, Haines et al. used a design thinking approach to promote implementation and effectiveness of a care coordination intervention for young adults with cancer, concluding that the approach helped harmonize evidence-based practices, contexts, and implementation strategies so that the intervention and its delivery were best designed to fit the implementation context (20).

Whereas design thinking concepts have been applied in business, engineering, social services, and health care (21), training in and use of this approach is relatively new in public health. Evidence is accumulating, however, to suggest design thinking’s utility for strategizing the application of evidence-based public health interventions. For example, design thinking has been used to develop mobile health interventions, (22) to promote healthy eating and physical activity in schools, (23, 24) and has been integrated with community engagement to address violence-related health disparities among Latino youth (25) and to ideate potential solutions to increase neighborhood park use (26). The combination of design thinking and implementation science approaches has been promoted for its potential to improve translation of evidence-based interventions into real-world settings (27). It has been suggested that design thinking methods can be used to address difficulties encountered during such translation (28), and to consistently operationalize implementation strategies (29).

As a human-centered approach, design thinking provides public health researchers and practitioners a systematic way to engage communities in co-producing solutions that align with community-identified needs, values, preferences, and assets. In other words, design thinking is set up to develop a contextually specific solution that solves the user’s “pain point” (30). For example, design charettes (i.e., collaborative workshops) are commonly used to engage participants to empathetically explore end-user experiences in order to tailor implementation strategies to the specific context of each community. The empathic, solution-oriented process of design thinking can also be effective in fostering social relationships within community groups, which contributes to the effectiveness of such groups in activating and sustaining social infrastructure (31).

Though more research is needed, the growing body of literature suggests that design thinking allows for more innovative, strategic, and contextually tailored intervention designs, which may increase participant adoption and maintenance of health behaviors. Furthermore, iterative problem solving associated with design thinking could support ongoing adaptation of evidence-based interventions to promote intervention sustainability in dynamic contexts.

## 3 Addressing complexity with systems science

Systems science is a broad term referring to the scientific understanding of complex adaptive systems, (32) as well as a set of tools to study the behavior of complex systems with applications ranging from developing theory to forecasting outcomes of interventions and informing policy. Systems science techniques are well-suited to examine dynamic, multi-level, and non-linear relationships and feedback loops that exist within evidence-based interventions and implementation contexts and strategies, making systems science a promising support for implementation sustainment and scale-up (33, 34).

The focus of implementation science during the past decade has shifted toward studying how to accelerate translation of evidence-based interventions into policy and practice, considering the complex, adaptive systems in which such interventions are implemented (9). The introduction of systems thinking as an effort to bring insights from systems science to implementation science challenges the thinking that implementation is a simple linear process, or that participatory research in itself will improve the use of evidence-based interventions in practice and policy settings (35). The use and sustainability of an evidence-based intervention is dependent on multiple, multi-level, dynamic processes (35, 36). The implication of this transition to systems thinking is that scientists must consider how to move their methods of investigation beyond a reductionist, isolated focus on a single part of a system toward a more holistic view (9).

Systems science includes informal causal mapping, mapping of social networks, and plotting of spatial relationships, as well as more formal mathematical modeling and computer simulation. Causal mapping and formal modeling with computer simulation are used to study system components and their dynamic interactions at multiple levels to better understand the behavior of complex systems (37). System science also includes methods for engaging actors directly in conceptualizing problems and participating in the development, interpretation, and transfer of ownership of results (e.g., soft systems methodology, participatory systems modeling, group model building, and community-based system dynamics) (38, 39).

The goals of participatory systems science approaches are to build a common vocabulary and agenda to describe a complex problem and its drivers, design potential solutions, and garner buy-in to implement the solutions that rise to the top after a range of options have been assessed with quantitative modeling approaches (40). For example, participatory systems science approaches have been applied to help community stakeholders in the HEALing Communities Study-New York State (HCS-NY) develop a shared understanding of the opioid crisis in order to inform local strategies for prevention and treatment (41). Community stakeholders who participated in the New York effort reported that the causal mapping approach helped them see the interconnectivity of complex factors, actors, and sectors and appreciate the need for multiple, mutually reinforcing strategies to avert opioid overdose and fatality.

Formal modeling with simulation methods have been used in public health research to increase the rigor of understanding complexity underlying public health challenges and to uncover novel ways to intervene in the system to solve real-world problems (4). Computer simulation modeling approaches are also used to test the potential impact of policies or interventions through simulations in which researchers create a virtual representation of a system (1). The computer simulation plays out the anticipated behavior of the system based on the input parameters, which can be changed to mirror various potential scenarios. This is particularly useful for interventions and policies that are time-consuming, expensive, and/or infeasible to test in controlled trials or in real-world settings.

The knowledge generated from such systems modeling can inform research and policy decision making, such as helping policymakers leverage an ideal combination of interventions to create the most impact or prioritize use of limited resources, as well as identifying potential unintended consequences of a modeled intervention. Unintended consequences may arise because complex systems are governed by feedback loops, time delays, and process of accumulation, which are not fully understood and usually ignored when designing interventions (1). This has important implications that need to be considered *a priori*, such as how to combine and sequence intervention strategies (14).

## 4 Integrating design thinking and systems science

Design thinking or systems science approaches can be used on their own, but embedding systems science approaches into the design thinking process can enhance design thinking’s usefulness as a framework to facilitate an iterative process toward systems change. Thus, certain systems science methods can enhance problem definition, while others can support the prototyping and testing phases.

If design thinking methods are most helpful in ‘packaging’ strategies (tools, devices, procedures) that will help achieve a desired objective or goal, then systems science methods are most helpful in defining and testing, via simulation, a theory of change, expressed as an overarching dynamic hypothesis. For example, again with reference to the HCS-NY, system dynamics modeling explains simulated trends in overdose fatality as a function of core feedback loops (structures). In turn, purposeful scenario analyses demonstrate how and when a specific (future) goal could be reached over time, given sufficient resources to support access to evidence-based harm reduction and treatment capacities. In this manner, the system dynamics model helps inform how to get ‘from A to B,’ and reveals key trade-offs and/or unintended consequences associated with achieving (and sustaining) such a goal. Thus, systems science methods model a problem (drivers of the status quo, or base case) and its associated solution space (potential goals), which calls for design thinking methods to ideate and prototype how to effectively achieve a goal.

**Figure 2:**
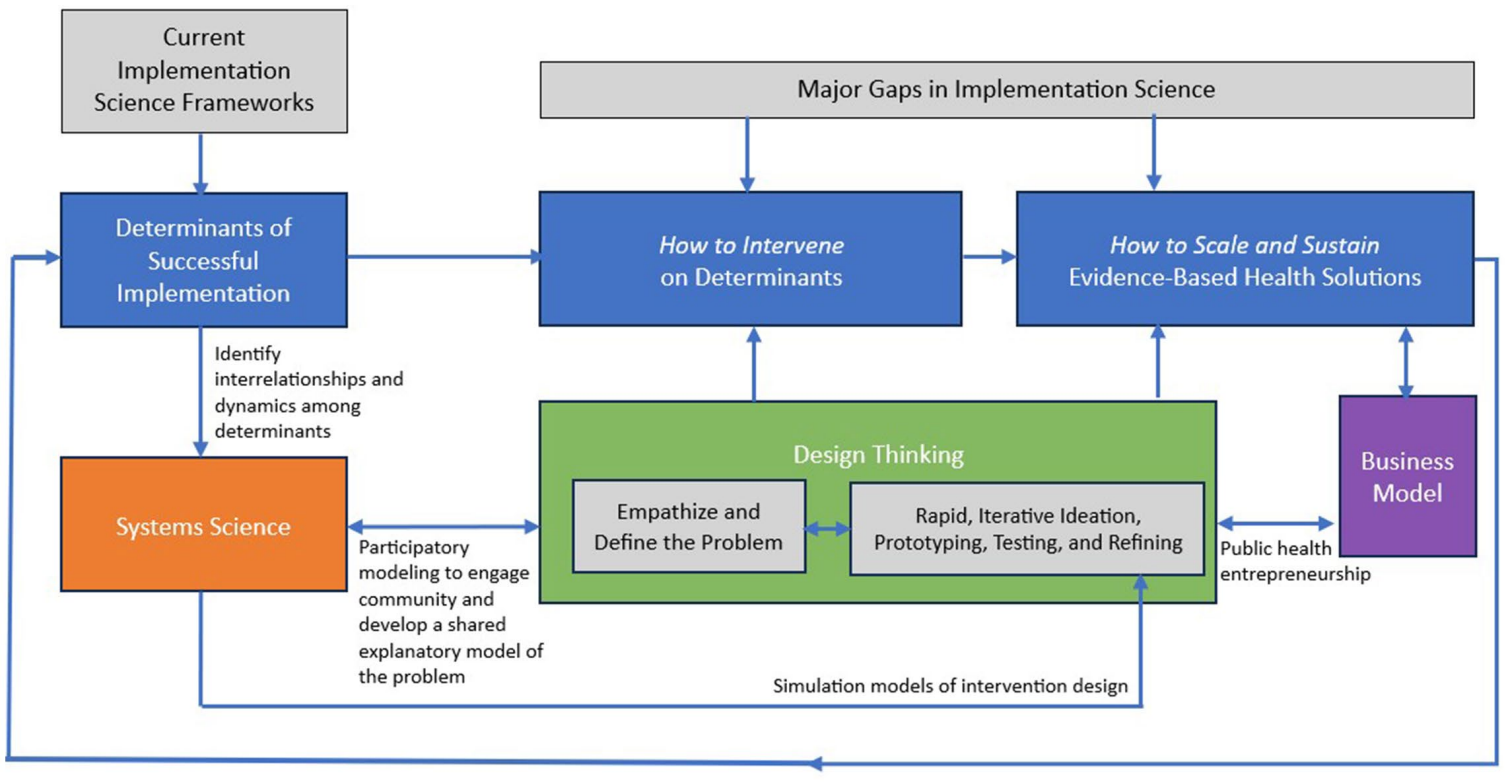
Leveraging systems science and design thinking to advance implementation science. Source: developed by T. T-K. Huang for this manuscript. In practice, it is an iterative process to bring together systems science, design thinking, and implementation science. As iterations occur both within each approach as well as collectively, the learnings immediately feed back into other parts of the process.

Figure 2 describes how systems science approaches could fit into a design thinking framework that aims to fill gaps in implementation science—namely, how to intervene on determinants of successful implementation and how to sustain and scale evidence-based public health solutions. For example, the systems science method of participatory modeling to engage communities and develop a shared model of a problem can serve design thinking’s first phase of empathizing and defining the problem, as in the HCS-NY State example described earlier. Systems science can also serve the experimentation and rapid prototyping process in design thinking by applying simulation modeling methods to test potential interventions. For example, applications of systems modeling approaches to improve food environments in Baltimore, Maryland included use of agent-based models—which use computer simulation to study complex systems from the ground up by examining how individual elements of a system (agents) behave as a function of individual properties, their environment, and their interactions with each other—to simulate the effects of placing warning labels on sugar-sweetened beverages in different combinations of grocery stores, corner stores, schools, and other settings where the beverages are available. The model estimated the potential effect of the labels on purchase and consumption of sugar-sweetened beverages (42).

Figure 2 also shows business modeling as an integral input to the process of sustaining and scaling solutions. This is because to move toward market feasibility, a viable and scalable model of revenue generation and cost-effective operation identified through the design thinking process must accompany the solution-oriented innovation. Business modeling entails defining the value proposition of a proposed public health solution, key inputs (e.g., partners, resources, and activities), customer base and segments, go-to-market strategies, and a revenue model. Even not-for-profit organizations must generate resources to achieve sustainability beyond traditional grant mechanisms.

## 5 Discussion

We urge three actions for the field of public health to help promote effective implementation, sustainment, and scaling of evidence-based public health solutions.

First, we must move toward use of a solution-oriented paradigm when approaching public health challenges. The public health community is generally organized around the problem-solving paradigm driven by scientific method, which is marked by a hypothesis-driven, linear, and often top-down approach to both problem solving and intervention design. This paradigm is well-suited for identifying drivers of health or disease but has less utility in determining *how* to intervene on these determinants, let alone sustain and scale an intervention, in an innately dynamic and complex system.

Second, we need more rigorous research and engagement in cross-disciplinary collaboration to test best practices for incorporating design thinking and systems science approaches into the implementation of community-engaged public health interventions. A recent systematic review concluded that few published peer-reviewed studies exist that use systems thinking and implementation science for designing and delivering population health interventions (43). Furthermore, we are not aware of any examples of empirical research that integrates all three—systems science, design thinking, and implementation science—in service of optimizing future intervention design and delivery. Documenting future efforts and assessing how these approaches facilitate and enhance intervention implementation can support development and dissemination of tools and training in best practices.

Third, design thinking and systems science curricula should be routinely incorporated into public health education. Design thinking is not a standard subject domain or practice method taught in public health schools, (44) and systems thinking—although now listed as a competency of graduate programs accredited by the Council on Education for Public Health (CEPH)—is not addressed in adequate depth in most public health programs. Interest is emerging in offering design thinking and entrepreneurship training in public health curriculum to teach future public health professionals how to create and apply business models, among other skills (such as marketing, finance, and business development and operations) that are important to scaling public health solutions (45–47). Such exposure to other disciplines can help set the stage for public health professionals to reach across disciplines and work with diverse collaborators, including those in the private sector.

In conclusion, the magnitude and persistence of current public health challenges demands creativity, innovation, and market feasibility. Most implementation science frameworks are top-down, deterministic, and linear, leaving gaps in understanding of both how to intervene on determinants of successful implementation and how to sustain and scale evidence-based solutions. Design thinking provides a deliberate, replicable process for intervention implementation and scaling that may increase acceptability and effectiveness of public health interventions by actively engaging communities in the design process and rapidly iterating innovation prototypes to maximize success. Systems science provides processes for understanding and managing complexity as well as testing assumptions and intervention ideas. A new paradigm for transformative change in public health is the integration of a design thinking approach with systems science and implementation science to promote more effective implementation, sustainment, and scaling of public health innovation.

## Data availability statement

The original contributions presented in the study are included in the article/supplementary material, further inquiries can be directed to the corresponding author.

## Author contributions

TH: Writing – review & editing, Conceptualization, Funding acquisition, Supervision. EC: Writing – review & editing, Writing – original draft. EH: Writing – review & editing. CH: Writing – review & editing. DS: Writing – review & editing. DL: Writing – review & editing. NS: Writing – review & editing. PH: Writing – review & editing.
